# Biocontrol strategies for fungal diseases of *Ganoderma lucidum*: from antagonistic microbes and synthetic communities to intelligent technologies

**DOI:** 10.3389/fmicb.2026.1813967

**Published:** 2026-05-12

**Authors:** Xianchao Chang, Xiaohan Jiang, Kexin Wang, Hui-lian Xu, Dongjie Chen, Mengmeng Kong, Xiaoyong Liu

**Affiliations:** 1 School of Biological Science and Technology, University of Jinan, Jinan, China; 2 National Engineering Research Center for Agricultural Products Logistics, Shandong Institute of Commerce and Technology, Jinan, China

**Keywords:** *Ganoderma lucidum*, biocontrol, fungal diseases, synthetic microbial community, antagonistic microorganisms, sustainable agriculture, artificial intelligence

## Abstract

*Ganoderma lucidum*, a rare macrofungus renowned for its substantial medicinal and economic significance, is increasingly at risk from various pathogenic fungi, such as *Neurospora sitophila*, *Trichoderma* spp., and *Fusarium graminearum*, particularly during extensive cultivation processes. Conventional chemical control strategies raise apprehensions concerning pesticide residues and environmental contamination, which contradict the principles of green and organic cultivation of *G. lucidum* alongside its medicinal safety prerequisites. As a result, the formulation of environmentally sustainable, efficient, and targeted biocontrol approaches has become a pivotal challenge for the sustainable progression of the *G. lucidum* sector. This review comprehensively examines the infection biology and pathogenic mechanisms associated with the primary pathogens that impact *G. lucidum*, while also highlighting the shortcomings of existing control measures. It emphasizes biological control tactics, which include the direct inhibitory impacts of antagonistic microorganisms, the establishment of synthetic microbial communities exhibiting synergistic effects, and integrated strategies utilizing precision-targeted genetically modified strains. This review focuses on the use of beneficial microorganisms (biocontrol agents) to protect *G. lucidum* from fungal pathogens, rather than using *G. lucidum* itself as a biocontrol agent. Ultimately, we propose a prospective research framework that amalgamates multi-omics technologies, nanobiotechnology, and artificial intelligence. This review aspires to create a solid theoretical basis and technical pathway for the development of a new generation of specialized biocontrol agents for *G. lucidum*, thereby propelling the cultivation of medicinal fungi toward greener, more precise, and more efficient agricultural methodologies.

## 1 Introduction

*Ganoderma lucidum*, which is also referred to as the “Mushroom of Immortality” or “Auspicious Herb” in Traditional Chinese Medicine, has been valued for over 2000 years and is described in ancient literature like the Shen Nong Ben Cao Jing as a high-grade medicinal substance. This much-revered fungus is known to have numerous health benefits. It is traditionally believed to modulate mental state, replenish vital energy (Qi), strengthen tendons and bones, and improve skin health (Wachtel-Galor et al., 2011). Modern pharmacological studies have confirmed that the major bioactive components of *G. lucidum*—including polysaccharides, triterpenoids, nucleotides, and sterols—exhibit a wide range of pharmacological effects. These effects include immunomodulatory, antitumor, antioxidant, anti-inflammatory, hepatoprotective and neuroprotective properties (Fang et al., 2025; Wang et al., 2025; Shi et al., 2025). *G. lucidum* has significant medicinal value and growing market demand, and its commercial cultivation has expanded rapidly. Consequently, the industry has become one of the core pillars to increase farmers’ income and support agricultural development in many regions (Kaushik et al., 2025).

Nevertheless, high-intensity and high-density farming has brought about a significant challenge of diseases that have become a bottleneck for the production, quality, and economic yield of *G. lucidum*. The optimal growth conditions (warm, humid, and rich in organic content) also promote the multiplication of other pathogenic microorganisms. Among the various pathogens, *Neurospora sitophila*, *Trichoderma* spp. (especially *T. viride* and *T. harzianum*), and *Fusarium graminearum* have been identified as the most dangerous (He et al., 2025; Xie, 2013). These pathogens are highly virulent and propagate rapidly, preventing mycelial development of *G. lucidum* and inhibiting primordial formation. Moreover, they can cause malformations or rotting of the fruiting bodies by competing for nutrients and space, releasing enzymes to break down cell walls, or producing mycotoxins, resulting in severe economic losses (Xie, 2013; Nur-Nazratul et al., 2021; Shi et al., 2020; Shi et al., 2021).

In the past, chemical pesticides were the most common approach for managing diseases affecting *G. lucidum*. Nevertheless, chemical pesticides have a number of disadvantages. To begin with, pesticide residues are incompatible with the medicinal and edible properties of *G. lucidum* and its safety standards, which may compromise the integrity of its bioactive compounds and the overall quality of products (Ji et al., 2013). Secondly, long-term and exclusive use of chemical pesticides may result in the development of resistance among pathogens, reducing the effectiveness of control measures over time (Ji et al., 2013). Furthermore, contamination of the cultivation environment by pesticides can negatively impact non-target organisms, such as beneficial soil microbiota, and this is not consistent with the principles of green and sustainable development that are globally accepted (Ji et al., 2013). Although physical control methods like high-temperature sterilization and ultraviolet irradiation are comparatively safe, they usually require high costs and high energy consumption and are mainly preventive, being ineffective against existing infections (Li et al., 2011).

Moreover, the occurrence of diseases not only causes short-term losses in yield but also greatly hinders the accumulation of medicinal compounds. Research shows that the contents of important bioactive substances, such as *G. lucidum* polysaccharides and triterpenoids, may decrease by 20–50% in fruiting bodies infected by pathogens, probably due to pathogen metabolism and plant stress responses. In areas with high disease incidence, the yield of *G. lucidum* fruiting bodies may decrease significantly. This situation not only endangers consumer health but also presents a great threat to the downstream traditional Chinese medicine industry. Therefore, it is necessary to develop efficient and specialized green control technologies to manage *G. lucidum* diseases, a critical and urgent task for the sustainable and healthy development of the industry (Shi et al., 2021; Wang et al., 2021).

Biological control, the use of beneficial microorganisms (biocontrol agents) or their metabolites to suppress pathogens and regulate the micro-ecological environment, has become a favored alternative to reduce or replace the use of chemical pesticides. This strategy is especially advantageous because it has high environmental compatibility, a low risk of inducing resistance, and the potential to promote the growth and yield of *G. lucidum* fruiting bodies. In addition, it provides an essential pathway to realize organic cultivation methods and support high-quality development in the *G. lucidum* sector (Syed Ab Rahman et al., 2018). Although biocontrol agents, most notably *Bacillus subtilis*, have shown significant efficacy in controlling diseases affecting various field crops, fruits, and vegetables (Syed Ab Rahman et al., 2018; Gerbore et al., 2014), the creation of specialized biocontrol agents that are both highly effective and stable against the main pathogens of *G. lucidum* remains underexplored, with few related products currently on the market (Li et al., 2011; Rincón et al., 2026).

## 2 Biological aspects, pathogenic mechanisms, and control challenges of major Ganoderma lucidum pathogens

### 2.1 Neurospora sitophila

*Neurospora sitophila* is a very fast-growing contaminant fungus classified in the *Ascomycota phylum*. It has a fluffy and loose mycelial structure and produces a large number of oval or almost spherical conidia that, when clustered together, have an orange-red color. *N. sitophila* spores are tiny and lightweight, which enables them to spread over long distances by air currents, human activities, and tools. When these spores infiltrate improperly cooled substrates or unsterilized cultivation bags, they germinate rapidly and outcompete the *G. lucidum* mycelium (Table 1).

**Table 1:** Summary of biological characteristics, pathogenic mechanisms, and control challenges associated with major *G. lucidum* pathogens.

Pathogen	Scientific name	Taxonomic group	Main infection characteristics	Key mycotoxins/metabolites	Impact on *G. lucidum*	Conventional control	Major challenges
Red bread mold	*Neurospora sitophila*	Ascomycota	Orange-red spore masses; rapid airborne dispersal; vigorous competition for nutrients	None reported (primarily competitive)	Mycelial growth cessation; entire bag loss	Environmental disinfection; thiophanate-methyl sprays	Extremely rapid transmission; inadequate chemical control effectiveness; potential for total crop failure
Green mold	*Trichoderma* spp. (*T. viride, T. harzianum*)	Ascomycota	Colonies shift from white to green; prevalent on substrate surface; mycoparasitism	Trichodermin; various volatile organic compounds	Mycelial inhibition; fruiting body malformation	Localized lime water treatment; carbendazim	Traditional agents may induce phytotoxicity; limited control duration; fungicide resistance
Fusarium rot	*Fusarium graminearum*	Ascomycota	Cotton-like mycelium; may develop pigmentation; produces multiple mycotoxins	DON, NIV, T-2, HT-2, ZEN, fumonisins	Mycelial growth inhibition; toxin accumulation in fruiting bodies	Environmental management; pydiflumetofen	Toxins pose product safety risks; residue concerns; resistance development

*N. sitophila* is primarily a competitor with *G. lucidum* mycelium for important nutrients, especially carbon and nitrogen sources in the substrate. Infected culture bags frequently have openings that are fully surrounded by vigorous orange-red mycelium and spore clusters, a condition often referred to as “red bread mold.” The active growth of *N. sitophila* mycelium tends to exceed that of *G. lucidum*, causing the latter to change color to yellowish-white, leading to growth cessation and eventually making the entire cultivation bag unsuitable (Xiong, 2015). Moreover, *N. sitophila* spore clusters disperse freely and abundantly with little interference (e.g., walking, ventilation), causing cross-contamination in the cultivation facility and creating a vicious cycle where one infected bag can ruin an entire growing room.

**Figure 1:**
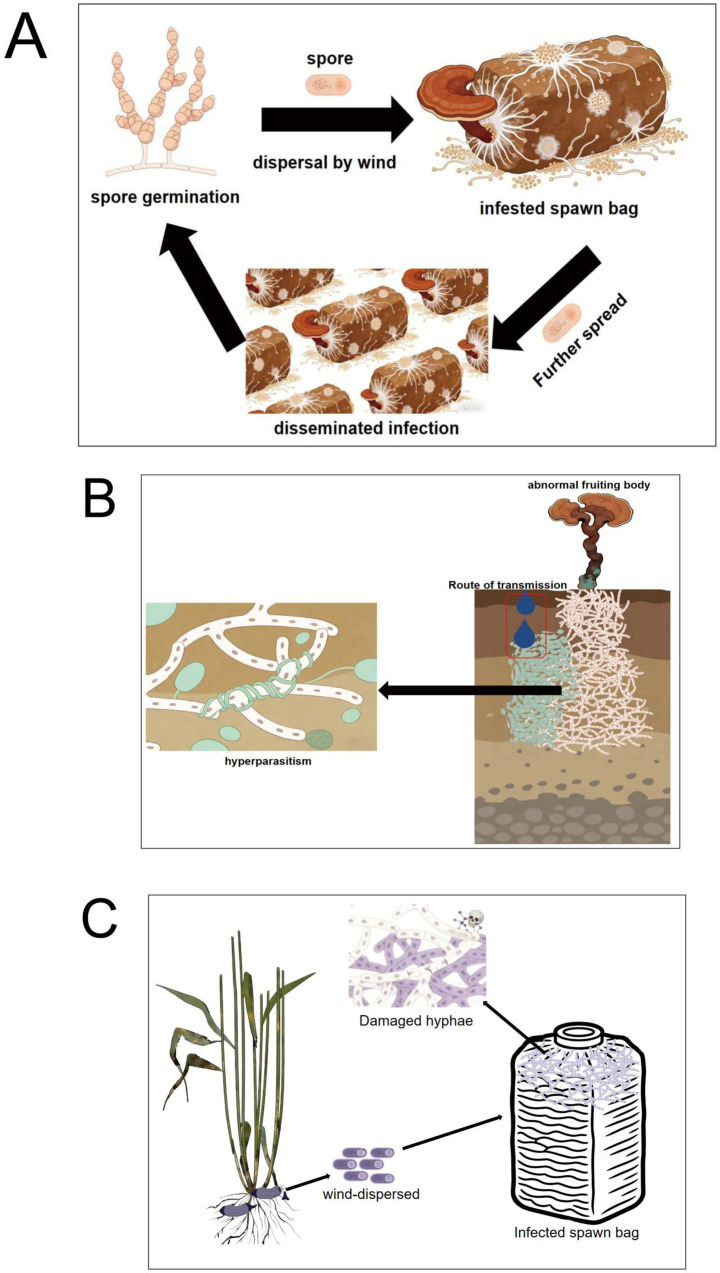
A schematic representation of the infection cycle and the damage inflicted by primary pathogens of *G. lucidum*. Panel **(A)** is the infection pathway of *Ganoderma lucidum* by *Neurospora*, panel **(B)** is the infection pathway of *Ganoderma lucidum* by *Trichoderma*, and panel **(C)** is the infection pathway of *Ganoderma lucidum* by *Fusarium graminearum*.

The existing control measures for *N. sitophila* are largely preventive in character. These strategies involve high cleanliness standards in cultivation sites and their surroundings, complete sterilization of substrates, proper cooling, and regular spraying of the environment with agents such as lime water, benzimidazole, or thiophanate-methyl (Xiong, 2015). Nonetheless, when an infection has already developed, chemical agents tend to have difficulty penetrating the thick spore clusters to reach the inner mycelium, meaning they are not very effective at controlling the infection. In cases of severe infection, the only options often require drastic actions like isolation, deep burial, or incineration, resulting in significant economic losses. Therefore, developing biocontrol agents that can colonize the substrate and prevent *N. sitophila* spore germination before it occurs is essential to overcome this problem (Figure 1).

### 2.2 Trichoderma spp.

*Trichoderma* spp. are fungi commonly encountered in soil and organic matter. The most common pathogenic species in *G. lucidum* cultivation include *Trichoderma viride* and *Trichoderma harzianum* (Wang et al., 2024; Xie and Tan, 2015). Initially, *Trichoderma* colonies are white but later turn green due to the production of large numbers of conidia (Wang et al., 2024).

**Figure 2:**
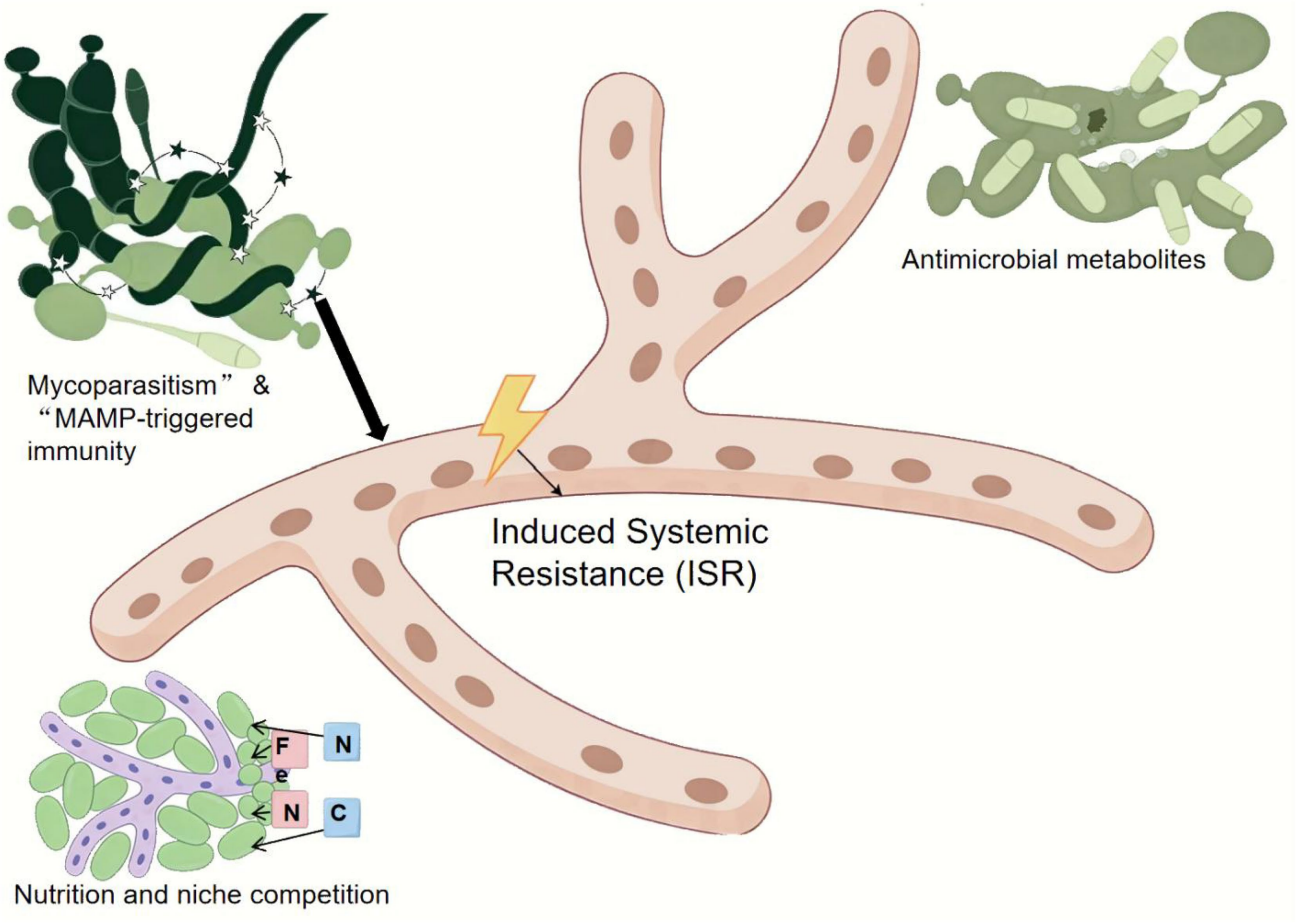
Schematic representation of the principal mechanisms of action of biocontrol agents against diseases affecting *G. lucidum*.

*Trichoderma* has a detrimental effect on *G. lucidum* through multiple mechanisms, making it a strong competitor. First, the rapid growth of *Trichoderma* mycelium enables it to quickly occupy the surface and interior of the substrate, depriving *G. lucidum* mycelium of nutrients and thus inhibiting its growth. Second, some *Trichoderma* species can identify, surround, and penetrate the hyphae of *G. lucidum* through mycoparasitism. This is achieved by excreting cell wall-degrading enzymes such as chitinases (e.g., Ech42), glucanases (e.g., Gluc78), and cellulases, which facilitate nutrient absorption (Wang et al., 2024). Third, *Trichoderma* produces various antimicrobial metabolites, such as trichodermin (a trichothecene mycotoxin) and volatile organic compounds (VOCs), which directly inhibit or kill *G. lucidum* mycelium. Finally, although some *Trichoderma* strains have been reported to trigger induced systemic resistance (ISR) in plants, the interaction between *Trichoderma* and *G. lucidum* is more complex and usually leads to strong antagonism (Wang et al., 2024) (Figure 2).

To effectively control *Trichoderma* contamination in *G. lucidum* cultivation, strategies that focus on early detection and localized intervention are required. The appearance of green mold spots during mycelial growth can be addressed by scrubbing with concentrated lime water, sealing with lime paste, or applying chemical treatments (Xie and Tan, 2015). However, infected fruiting bodies should be removed as soon as possible. Although chemical fungicides such as carbendazim and chlorothalonil show some level of effectiveness, studies indicate that these agents strongly inhibit the mycelial growth of *G. lucidum*, leading to toxicity (Xie and Tan, 2015). Moreover, the growing resistance of *Trichoderma* species, particularly *T. viride* and *T. harzianum*, to commonly used fungicides is a serious problem. Isolates obtained from infected Agaricus bisporus cultivation bags have shown reduced sensitivity to iprodione and extensive resistance to trifloxystrobin. This resistance is likely due to *Trichoderma*’s strong detoxification metabolism and the protective role of its cell wall (Kosanović et al., 2015).

Under such limitations, the creation of specific biocontrol agents is extremely important. Promising approaches include identifying highly specific antagonists that can inhibit *Trichoderma* without harming *G. lucidum*, or using non-pathogenic *Trichoderma* strains (e.g., particular commercial preparations of *T. harzianum*) to outcompete pathogenic fungi. Recent studies highlight the promise of novel mechanisms, including peptide antibiotics produced by *Bacillus amyloliquefaciens* SQR9, which disrupt the integrity of resistant *Trichoderma* cell membranes, leading to the release of cellular contents (Xu et al., 2014). In pot experiments, this strategy achieved control efficacy up to 77%, representing a viable alternative to traditional fungicides.

### 2.3 Fusarium graminearum

*Fusarium graminearum* is recognized as a significant plant pathogenic fungus worldwide and has been primarily associated with *Fusarium* head blight (FHB) of cereal crops. This pathogen leads to substantial yield losses and contamination of grains with harmful mycotoxins including deoxynivalenol (DON) and nivalenol (NIV) (Palacios et al., 2021). Recently, it has also been identified as a severe pathogen in *G. lucidum* production (Kong et al., 2023).

*F. graminearum* has strong saprophytic competitive power, enabling it to colonize cellulose-rich substrates very quickly. Its mycelium is cottony in texture, with colors ranging between white and pale red, and it competes vigorously for resources. The production of mycotoxins is a particularly worrisome characteristic of *F. graminearum*. This fungus releases various trichothecene mycotoxins including deoxynivalenol (DON, also known as vomitoxin), nivalenol (NIV), T-2 toxin, HT-2 toxin, and zearalenone (ZEN), as well as fumonisins (FB1, FB2) (Ben Salah-Abbès et al., 2021). These mycotoxins are dangerous in two ways: they can directly harm *G. lucidum* mycelium by preventing the synthesis of cell wall components, leading to a significant reduction in growth rates; moreover, they can accumulate in *G. lucidum* fruiting bodies, posing health risks to humans and animals through the food chain. Chronic exposure has been associated with immunosuppressive effects, neurotoxicity, and reproductive disorders in various toxicological studies (Zhu et al., 2020).

Control of the cultivation environment, especially the maintenance of appropriate temperature and humidity, is critical for preventing *F. graminearum* infection. Once infection has been identified, corrective actions can be taken, including application of alcohol to the contamination point or use of lime water. More severe cases may require emergency measures with newer fungicides such as pydiflumetofen (a strong succinate dehydrogenase inhibitor) or chlorine dioxide. However, chemical control approaches also have drawbacks, including possible residue accumulation, resistance development, and limited efficiency in toxin elimination. The problem is exacerbated by the high stability of mycotoxins produced by *Fusarium*, including DON, in cultivation substrates. These long-lasting toxins are resistant to normal physical and chemical remediation processes, thus preventing the development of *G. lucidum* mycelium and accumulating in fruiting bodies. Therefore, such toxins can enter food and medicinal supply chains even after eradication of toxigenic strains (Lu et al., 2022).

An ideal biocontrol strategy should not only prevent the pathogen but also effectively break down these toxins. One promising approach is to use microorganisms such as *Sphingomonas* sp. KSM1, which can enzymatically transform DON into less toxic or non-toxic byproducts using their P450 monooxygenase system (Ito et al., 2013). Future studies should focus on combining such effective detoxifying strains with antagonistic strains against *Fusarium* to establish functionally complementary synthetic microbial communities (SynComs), thereby providing a more sustainable and holistic method for controlling *Fusarium* and mycotoxin contamination (Table 2).

**Table 2:** Antagonistic species against *G. lucidum* pathogens: mechanisms and efficacy.

Antagonist type	Representative species/strain	Target pathogen	Main mechanism of action	Efficacy (lab/field) key	References
Bacteria	*Bacillus amyloliquefaciens* TS-1203	*Trichoderma* spp.	Lipopeptide secretion (surfactin, iturin, fengycin); nutrient competition	65–78% field efficacy	Hou et al. (2017)
Bacteria	*Pseudomonas aeruginosa* 2016NX1	*Fusarium* spp.	Siderophore-mediated iron chelation; phenazine antibiotics	70–85% lab efficacy	Liu et al. (2020)
Bacteria	*Bacillus subtilis* + *Pseudomonas fluorescens* (SynCom)	*N. sitophila*	*Trichoderma*, *Fusarium* multi-strain synergy; niche occupation; antimicrobial secretion	90% lab efficacy	Du et al. (2025), Jia et al. (2023)
Yeast	*Pichia anomala*	*Fusarium* spp.	Competition for space and nutrients; biofilm formation	54% lab efficacy	Laitila et al. (2007)
Fungus	*Talaromyces pinophilus* HD25G2	*Fusarium culmorum* (related to *F. graminearum*)	Mycoparasitism; iron competition; cell wall-degrading enzymes	72% pot efficacy	Bennacer et al. (2025)

## 3 Biocontrol approaches for *Ganoderma lucidum* diseases: transitioning from isolated agents to integrated systems

### 3.1 Utilization of traditional antagonistic microorganisms

Antagonistic microorganisms are beneficial microbes that can inhibit pathogen proliferation through direct or indirect mechanisms. Among bacteria, *Bacillus* and *Pseudomonas* are the most widely researched genera for controlling *G. lucidum* diseases. *Bacillus* species offer the distinct advantage of producing highly resistant endospores, which facilitate industrial production, long-term formulation, and storage. They secrete a variety of lipopeptide antibiotics (surfactin, iturin, fengycin) that disrupt fungal cell membranes, compete effectively for nutrients and ecological niches, and can stimulate defense-related genes in *G. lucidum* mycelium via signaling molecules. A representative strain, *Bacillus amyloliquefaciens* TS-1203, exhibits 65–78% field efficacy against *Trichoderma* diseases (Hou et al., 2017). However, *Bacillus* typically requires higher cell densities to achieve rapid pathogen suppression compared to *Pseudomonas*. In contrast, *Pseudomonas* species are fast-growing and produce a different arsenal of antimicrobial metabolites, including siderophores that chelate iron (indirectly starving pathogens), pyocyanin, 2,4-diacetylphloroglucinol (DAPG), and hydrogen cyanide. Strain *Pseudomonas aeruginosa* 2016NX1 secretes phenazines with strong antifungal activity (Liu et al., 2020). The main limitation of *Pseudomonas* is its inability to form durable endospores, making formulation and shelf-life more challenging. Nevertheless, *Pseudomonas* is often co-applied with *Bacillus* to achieve synergistic effects, leveraging the fast action of the former and the persistence of the latter. Beyond bacteria, yeasts and non-pathogenic fungi offer complementary advantages. Yeasts such as *Pichia anomala* and *Candida oleophila* compete for space and nutrients, form biofilms, and produce volatile organic compounds or exhibit mycoparasitism (Laitila et al., 2007; Freimoser et al., 2019). Filamentous fungal antagonists like *Talaromyces pinophilus* and *Clonostachys rosea* can actively overgrow and parasitize *Fusarium* and *Trichoderma* hyphae (Bennacer et al., 2025; Krauss et al., 2013). The primary drawback of fungal and yeast biocontrol agents is their slower growth compared to bacterial agents and greater sensitivity to environmental conditions (temperature, humidity). However, they often possess more diverse mechanisms of action and may establish longer-lasting associations with the substrate (Salerno et al., 2024). Although their application in *G. lucidum* cultivation is still in early stages, these organisms represent a valuable resource for future biocontrol development (Agirman et al., 2023).

### 3.2 Synthetic microbial communities

Although individual strains of beneficial microorganisms can show significant effectiveness under controlled laboratory conditions, their ability to reliably colonize and manage pathogens in complex field environments often varies. To solve this problem, Synthetic Microbial Communities (SynComs) are carefully constructed by combining different, strategically selected microorganisms in specific ratios to mimic the stability and functionality of natural microbial ecosystems.

SynComs form a strong, multi-layered defense system through task division and cooperation among microbial constituents. For example, an efficient SynCom for the biocontrol of *G. lucidum* could consist of: Strain A, which synthesizes antibiotics to directly fight pathogens; Strain B, which efficiently uses available carbon sources to fill ecological niches; Strain C, which forms biofilms to protect the community; and Strain D, which neutralizes toxins generated by pathogens. This collaborative strategy not only improves the community’s stability and broad-spectrum effectiveness but also reduces the chances of pathogens becoming resistant (Wang et al., 2023; Moussa and Iasur Kruh, 2025).

Laboratory studies have shown that a SynCom consisting of *Bacillus subtilis* and *Pseudomonas fluorescens* achieved control efficacy of more than 90% against complex diseases caused by *N. sitophila* and *Trichoderma* species, which is much higher than the performance of any individual strain treatment (Du et al., 2025; Jia et al., 2023). Additional examples further support the SynCom approach. For instance, a SynCom comprising *Bacillus subtilis*, *Pseudomonas fluorescens*, and *Streptomyces rochei* was shown to suppress *Fusarium wilt* in cucumber by more than 85%, with synergistic effects exceeding those of individual strains. Another study constructed a four-member SynCom from the maize core microbiome that reduced *Fusarium verticillioides* infection by 70%. These examples highlight the potential of multi-species consortia for robust disease suppression in medicinal fungus cultivation (Qin et al., 2025; Shao et al., 2025).

### 3.3 Genetically engineered strains

Recent advances in synthetic biology have provided powerful tools that can substantially enhance the functionality of biocontrol agents. Techniques such as CRISPR-Cas9 enable precise genetic modifications, allowing biocontrol strains to gain new or improved features (Zhang et al., 2021; García-Murillo et al., 2023).

The production of antimicrobial lipopeptides or polyketides by biocontrol agents can be significantly enhanced by amplifying key regulatory genes in antibiotic biosynthesis gene clusters (Li et al., 2025). Additionally, the introduction of antimicrobial genes from various microorganisms into a biocontrol agent can enhance its ability to suppress a wider range of pathogens (Li et al., 2025).

Precision Targeting of Pathogen Virulence Genes: Engineered strains can be designed to specifically target and silence essential virulence genes, including TRI5 in pathogens such as *F. graminearum*. As an example, engineered *B. subtilis* can secrete small RNAs that trigger RNA interference (RNAi); when ingested by the pathogen, these RNAs inhibit the toxin production pathway specifically (Vieira et al., 2021). The chemical fungicide pydiflumetofen, a succinate dehydrogenase inhibitor (SDHI), has been reported to reduce DON toxin biosynthesis in *F. graminearum* by more than 80%, primarily by suppressing the expression of the TRI5 gene and disrupting the trichothecene biosynthetic pathway (Huang et al., 2024).

Even though the prospects of genetically engineered strains are encouraging, there are still challenges in terms of biosafety, regulatory compliance for environmental release, and social acceptance. Future efforts must focus on improving environmental risk assessments and creating appropriate regulatory frameworks (Vieira et al., 2021; Leung et al., 2020).

### 3.4 Combined *Ganoderma lucidum*-microbe strategy

This emerging approach effectively combines the natural immune defense systems of *G. lucidum* with the antagonistic properties of biocontrol agents. The polysaccharides produced by *G. lucidum* are necessary active components that also act as Microbe-Associated Molecular Patterns (MAMPs). When biocontrol agents colonize around *G. lucidum* hyphae, their metabolic activities may trigger the release or production of polysaccharide fragments from *G. lucidum*. These fragments are then recognized by Pattern Recognition Receptors (PRRs) on the surface of *G. lucidum* hyphae, initiating a cascade of defense reactions. Such responses include enhanced production of reactive oxygen species (ROS), increased activity of defense-related enzymes such as peroxidase (POD) and phenylalanine ammonia-lyase (PAL), and strengthening of hyphal cell walls (Jones and Dangl, 2006; Pieterse et al., 2014). This prepares *G. lucidum* to become more resistant to future pathogen attacks. At the same time, biocontrol agents actively counteract pathogen tactics through direct competition and antagonistic interactions at the hyphal interface. This two-pronged approach of internal priming and external defense promotes a synergistic effect in disease management, where the overall impact is greater than the sum of the individual components. For example, a recent study demonstrated that co-application of *B. amyloliquefaciens* and *G. lucidum* polysaccharides reduced *Trichoderma* infection by 65% compared to a 40% reduction with the bacterium alone (Kumari et al., 2024; Harman et al., 2004; Ongena and Jacques, 2008).

## 4 Integration of technologies and future outlook: a new era of precise and intelligent biocontrol for *Ganoderma lucidum*

The combination of multidisciplinary technologies will greatly assist future developments in the biocontrol of *G. lucidum*. Scientists can enhance understanding and application of these technologies by developing a research framework that incorporates state-of-the-art innovations.

### 4.1 Multi-omics approaches for comprehensive analysis of biocontrol mechanisms

Metagenomics and culturomics allow scientists to directly isolate total DNA from *G. lucidum* cultivation systems and use high-throughput sequencing methods. This approach enables comprehensive determination of microbial community composition and the genetic potential of non-cultured beneficial microbes, which may be used to develop new biocontrol measures. Transcriptomics and proteomics reveal gene and protein expression patterns during interactions between *G. lucidum*, pathogens, and biocontrol agents, identifying key points in defense signaling pathways of *G. lucidum*. Moreover, these approaches elucidate the regulatory networks controlling antimicrobial compound production by biocontrol agents and stress response mechanisms of pathogens, thus improving our understanding of these complex interactions (Rao et al., 2022; Fatima et al., 2022). Metabolomics determines the key metabolites produced during these interactions, including antimicrobials, signaling molecules, and toxins, providing important information on the chemical nature of antagonistic substances. This methodology can be used to discover new molecular targets and interaction pathways, which may help in the development of novel biocontrol measures (Rao et al., 2022).

### 4.2 Amplifying agent effectiveness through nanobiotechnology and emerging functional materials

Nanomaterials have emerged as an important breakthrough for enhancing the stability and delivery efficiency of biocontrol agents, which are critical for plant disease control. These materials offer various functionalities that significantly increase the effectiveness of biocontrol strategies. Nanocapsules protect biocontrol agents and their metabolites from degradation by exposure to ultraviolet (UV) radiation, high temperatures, and extreme pH values. This defense mechanism ensures that biocontrol agents remain viable and effective for long periods even under adverse environmental conditions (Rajwade et al., 2020). The introduction of nanomaterials also allows the slow and long-term release of biocontrol agents or antimicrobial substances. This regulated release extends their duration of action, enabling longer-lasting effects against pathogens and reducing the number of required applications (Rana et al., 2025). Furthermore, surface modifications enable nanocarriers to deliver active ingredients precisely to pathogen-rich regions. This specific delivery increases the utilization efficiency of biocontrol agents and reduces the total application rates required, thus minimizing environmental impact and resource consumption (Choudhary et al., 2023). Such developments in nanotechnology not only enhance the efficiency of biocontrol agents but also offer an environmentally friendly and sustainable solution for plant disease control, lessening reliance on traditional chemical pesticides (Pasquoto-Stigliani et al., 2023).

Beyond these general nanocarrier platforms, recent studies have demonstrated that specific functional nanomaterials, particularlyFe₃O₄ nanoparticles (NPs), exhibit direct size-dependent antifungal activity against *Fusarium* pathogens and prime host immunity via ROS and SA/JA pathways (Kong et al., 2025a, 2025b, 2026a). Low-dose Fe₃O₄ NPs (0.5 mg/L) trigger metabolic reprogramming that avoids growth-defense trade-offsp (Kong et al., 2026b).

### 4.3 Artificial intelligence and large-scale models facilitating accurate prediction and decision-making

The large-scale integration of multi-omics data, environmental factors, and field efficacy measures provides a solid foundation for the application of artificial intelligence (AI) in biocontrol. AI is not merely a predictive tool but also a powerful means to understand and enhance biocontrol mechanisms.

Efficient Strain Screening: By using machine learning algorithms, researchers can predict the biocontrol effectiveness of microorganisms based on their genomic properties. For example, a random forest model was trained on genomic signatures (e.g., presence of non-ribosomal peptide synthetase genes) and achieved 93.5% accuracy (Ualiyeva et al., 2026). This methodology allows rapid discovery of highly effective strains from large strain collections, significantly shortening the research and development period (Yue et al., 2025; Liang et al., 2026). Furthermore, AI-driven frameworks have been developed to optimize biochar-based biocontrol carriers through digital twin simulations and real-time closed-loop control, enabling personalized formulation design for specific pathosystems (Kong et al., 2026a).

Intelligent SynCom Design: Deep learning approaches can study interaction patterns among various microbial combinations. A graph neural network was trained on transcriptomic data from *G. lucidum*–*Trichoderma*–*Bacillus* tripartite interactions, revealing that the biocontrol agent upregulates *G. lucidum*’s PAL and POD genes – key nodes in the defense signaling pathway. This ability helps predict optimal compositions and relative abundances in microbial communities, which is essential for the systematic design of SynComs. These methods reflect current trends in microbiome engineering, with AI being a key factor in developing more potent and versatile biocontrol strategies (Gómez-Lama Cabanás and Mercado-Blanco, 2025). Furthermore, AI-driven frameworks have been developed to optimize biochar-based biocontrol carriers through digital twin simulations and real-time closed-loop control, enabling personalized formulation design for specific pathosystems (Kong et al., 2025c).

Disease Prediction and Biocontrol Scheme Recommendation: A major application is the development of large-scale AI models (e.g., deep learning architectures with millions of parameters) that integrate historical meteorological data, real-time environmental sensor readings, pathogen monitoring information, and *G. lucidum* growth models. Reinforcement learning has also been used to optimize the ratio of three bacterial strains in a SynCom, leading to a 40% increase in siderophore production and enhanced competitive exclusion of *F. graminearum*. These models have the potential to predict disease risks and recommend the best types, combinations, timings, and dosages of biocontrol agents for specific cultivation conditions and developmental stages of *G. lucidum* (Gómez-Lama Cabanás and Mercado-Blanco, 2025; Oufensou et al., 2025).

### 4.4 Field promotion and system construction

To translate these technological advances into practical benefits, they must be deployed under real cultivation conditions. Several strategic approaches can promote these innovations successfully. Establishing demonstration bases in major *G. lucidum* growing regions (e.g., Anhui, Jilin provinces) is essential. These farms will serve as experimental facilities that rigorously test the practical efficiency and economic viability of innovative biocontrol agents and intelligent decision-support systems. The demonstration bases are expected to generate useful data that will facilitate the large-scale implementation of these advanced technologies. Research on application methods such as seed coating, substrate inoculation, and targeted drip irrigation using fermented biocontrol solutions should be given high priority (Barcenas-Giraldo et al., 2026; Song et al., 2026). Moreover, investigating materials that can increase the lifespan of biocontrol agents in soil or substrates is important for improving the effectiveness and sustainability of biocontrol measures in agricultural systems (Rajwade et al., 2020; Rana et al., 2025). To facilitate the implementation of biocontrol principles, it is essential to enhance technical training among producers. By providing extensive educational programs, growers will be better able to understand and implement biocontrol strategies. The ultimate aim is to establish an integrated green management system for diseases that attack *G. lucidum*. This system must include real-time monitoring, intelligent early warning, precise decision-making, and efficient control, thus ensuring a holistic approach to disease management (Pasquoto-Stigliani et al., 2023). The combination of these strategies is anticipated not only to drive the practical application of biocontrol technologies but also to promote sustainable agricultural practices and strengthen crop health.

## 5 Conclusion

The development of a high-quality *G. lucidum* industry is closely linked to overcoming disease-related challenges. Biocontrol is advancing beyond the use of single antagonistic microorganisms toward an era of integration and precision that includes the design of synthetic microbial consortia, genetic engineering, and combined immune strategies. Recent studies have emphasized the promise of biocontrol agents such as *Trichoderma atroviride* LZ42, which releases volatile organic compounds that promote plant growth while suppressing diseases like *Fusarium* wilt in tomato seedlings (Rao et al., 2022). Moreover, culture filtrates obtained from *G. lucidum* have shown significant nematicidal effects against *Meloidogyne incognita*, indicating their potential as eco-friendly biocontrol options (Fatima et al., 2022). To propel this field forward, future investigations should concentrate on several pivotal areas: comprehensive resource exploration employing multi-omics approaches to continuously investigate the distinctive microbial resource pool associated with *G. lucidum*; clarification of detailed mechanisms to understand the complex interaction networks among biocontrol agents, pathogens, and the host *G. lucidum* at both molecular and systemic levels; integration of interdisciplinary technologies encouraging the innovative application of nanotechnology, synthetic biology, and artificial intelligence within the biocontrol arena; and facilitating application and execution by converting cutting-edge laboratory discoveries into tangible field applications via demonstration initiatives and system development (Ning et al., 2026). Through the development of an intelligent, efficient, and flexible green control system for *G. lucidum*, we can guarantee the sustainable development of this traditional “Mushroom of Immortality,” thus securing the safety and efficacy of its products. Moreover, the technological models and knowledge acquired may be used in the production of other high-value medicinal mushrooms including *Cordyceps*, *Phellinus igniarius*, and *Wolfiporia extensa*. This approach has the potential to bring significant economic, ecological, and social benefits, bringing Chinese wisdom to the healthy growth of the global medicinal fungi industry and strengthening food security.
